# Clinical Outcomes and Prognosis Analysis of Younger Bladder Cancer Patients

**DOI:** 10.3390/curroncol29020052

**Published:** 2022-01-28

**Authors:** Mierxiati Abudurexiti, Jie Ma, Yao Li, Chuanyi Hu, Zhikang Cai, Zhong Wang, Ning Jiang

**Affiliations:** 1 Department of Urology, Shanghai Pudong New Area Gongli Hospital, Shanghai 200135, China; 17111230008@fudan.edu.cn (M.A.); mj01310@glhospital.com (J.M.); ly01663@glhospital.com (Y.L.); hcy01425@glhospital.com (C.H.); czk01684@glhospital.com (Z.C.); 2 Department of Urology, Naval Military Medical University Affiliated Gongli Hospital, Shanghai 200135, China; 3 Institute of Oncology, Fudan University Shanghai Cancer Center, Shanghai 200032, China; 4 Department of Urology, Shanghai Ninth People’s Hospital, Shanghai Jiao Tong University School of Medicine, Shanghai 200011, China

**Keywords:** bladder cancer, 40 years, prognostic factors, survival

## Abstract

Background: Generally, little is known about prognostic factors in bladder cancer patients under 40 years of age. We therefore performed a retrospective study to identify prognostic factors in these younger bladder cancer patients. Methods: We collected clinicopathological data on bladder cancer patients ≤40 years old diagnosed between 1975 and 2018 from the Surveillance, Epidemiology, and End Results (SEER) database. Survival curves were generated using the Kaplan–Meier method, and the differences between groups were analyzed using the log-rank test. Univariate and multivariate Cox hazards regression analyses were performed to define hazard ratios (HRs) for cancer-specific survival (CSS). Results: There were statistical differences in race, histological type, cancer stage, tumor size, and surgery treatment groups between overall survival and CSS. Only tumor size and cancer stage were significant independent prognostic risk factors in younger bladder cancer patients for the prediction of CSS. Conclusion: Tumors greater than 30 mm in size and a more advanced stage of bladder cancer were indicative of a poor prognosis in bladder cancer patients ≤40 years old, and long-term follow-up is suggested.

## 1. Introduction

With the number of smokers increasing, the incidence of bladder cancer is rising, and bladder cancer is currently the seventh most commonly diagnosed malignancy in males, while it is the tenth most common cancer worldwide [1]. The age-standardized incidence rate in males is 9.5 per 100,000, while in females it is 2.4 per 100,000 [1]. The age-standardized mortality rate is 3.3 per 100,000 in males and 0.86 per 100,000 in females [1]. However, the age-standardized incidence and mortality rate in younger patients (≤40 years old) with bladder cancer is 0.15 per 100,000 and 0.03 per 100,000, respectively [1]. 

Tobacco is the most important risk factor for bladder cancer, while chemical exposure is second [2]. Prognostic factors of bladder cancer are also being studied, and it has been found that age, tumor grade, race, American Joint Committee Cancer (AJCC) cancer stage, and tumor size are all independent prognostic factors in bladder cancer [3]. We previously analyzed a 12-gene signature used in predicting overall survival (OS) in patients with muscle-invasive bladder cancer [4]. Although the incidence and mortality rates are considerably lower in younger (≤40 years old) bladder cancer patients, prognostic clinicopathological characteristic factors in these patients are still unclear. 

In this study, we collected data on the clinicopathological characteristics of younger (≤40 years old) bladder cancer patients from the Surveillance, Epidemiology, and End Results (SEER) registry, and we analyzed the correlation of these characteristics with the survival and prediction of prognosis.

## 2. Patients and Methods

From the SEER database recorded between 1975 and 2018, we collected data on primary bladder cancer patients, who were ≤40 years old at diagnosis, where the sequence number-central codes were ”0,1”. A total of 3789 patients were included in this study. Because of incomplete documentation, not all patients were analyzed for each characteristic. Age, year of diagnosis, gender, race, and histologic type were documented in 3789 patients and were all used in the analysis. However, tumor size, surgical treatment, and household income were documented in only 408, 763, and 2364 patients, respectively. Because the cancer stage of each patient included in the study was made on the basis of the edition of the *AJCC Cancer Staging Manual* at the time of diagnosis, different AJCC editions were used in staging patients (3rd edition: 1008 patients; 6th edition: 772 patients; 7th edition: 378 patients; and 8th edition: 62 patients).

All characteristics were summarized using descriptive statistics, and differences among each subgroup were compared using one-way analysis of variance for continuous variables and the chi-square or Fisher’s exact test for categorial variables. Survival curves were generated using the Kaplan–Meier method, and the differences between each group were analyzed using the log-rank test. Univariate and multivariate Cox hazards regression analyses were performed to define hazard ratios (HRs) for cancer-specific survival (CSS).

All tests were two-sided, and statistical significance was set at *p* < 0.05. All statistical analyses were performed using SPSS statistics software version 22.0 (IBM Corporation, Armonk, NY, USA).

## 3. Results

**Figure 1:**
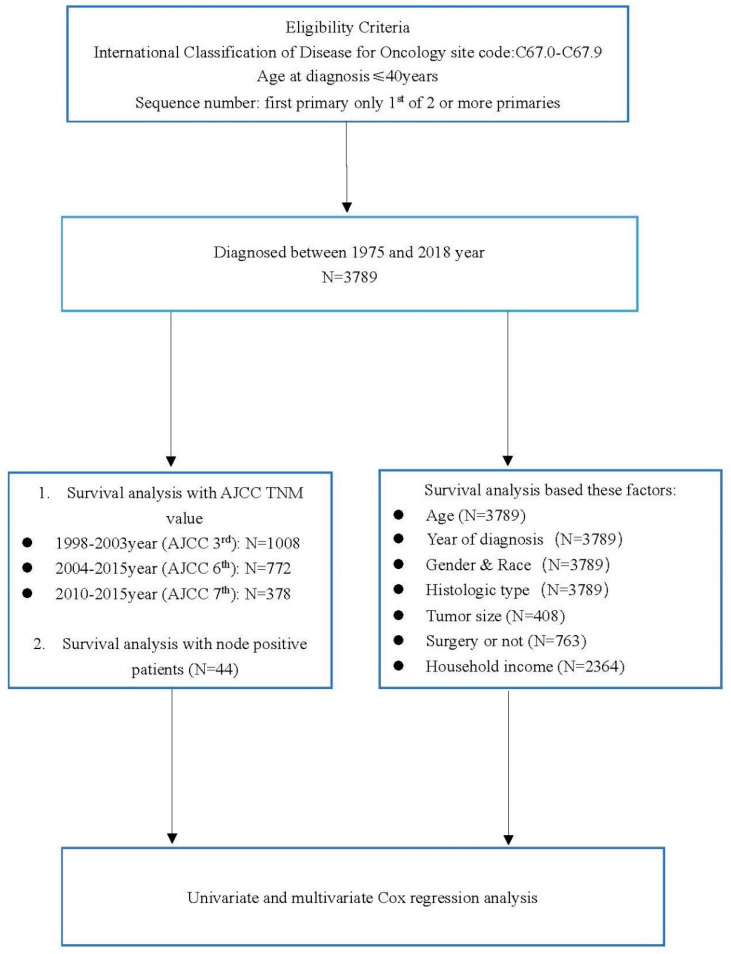
Flow chart showing selection and analysis process. AJCC = American Joint Committee on Cancer.

A total of 3789 primary bladder cancer patients ≤40 years old were diagnosed between 1975 and 2018, and the median age was 35 years. As shown in Figure 1 and Table 1, 3559 (93.93%) patients were older than 20 years and 2474 (65.29%) patients were diagnosed between 1975 and 2000. There were 2747 male patients and 1042 female patients, and the ratio of male to female was 2.7:1. There were 3266 (86.20%) Caucasians, who tend to have a high incidence of bladder cancer, included in this study. Most patients (3384 (89.31%)) had papillary carcinomas of the bladder, which was the main histological type. Nearly 89% of patients were diagnosed with early-stage cancer. The node-positive rate was calculated by the ratio of the number of positive nodes to the number of examined nodes, and 75% of patients were in the <50% node-positive group. There were 238 (58.33%) patients with tumors that were ≤30 mm in size, while in 170 patients, tumors were >30 mm. Most patients (92.27%) underwent surgery. Furthermore, 71.74% of patients had a median household income of ≥USD 65,000/year. There was no significant difference between each characteristic group. Five patients had liver metastasis and ten patients had lung metastasis; however, two patients had both liver and lung metastasis. Three (60%) patients died of cancer in the liver metastatic group, and the longest survival time was 11 months. Eight (80%) patients died of cancer in the lung metastatic group, and the longest survival time was 22 months. However, two patients who had liver and lung metastasis both died of cancer, and the longest survival time was 5 months.

**Figure 2:**
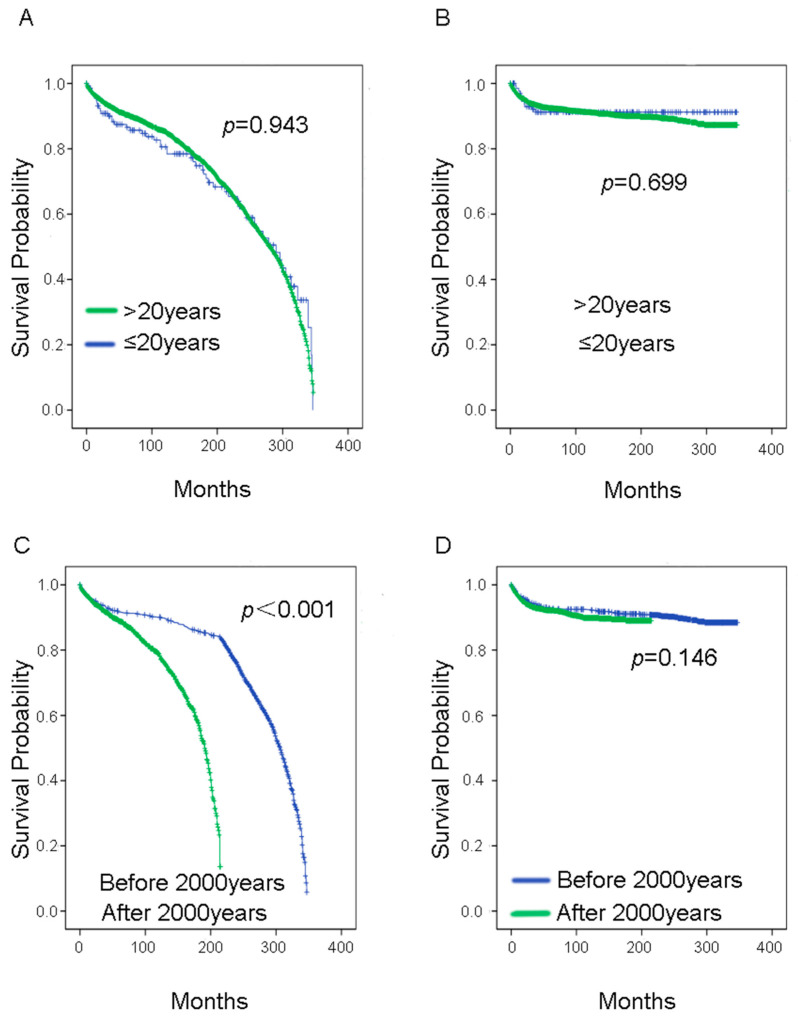
Survival analysis in younger bladder cancer patients at the age of diagnosis (**A**) OS, (**B**) CSS, and year of diagnosis (**C**) OS, (**D**) CSS. OS = overall survival; CSS = cancer-specific survival.

**Figure 3:**
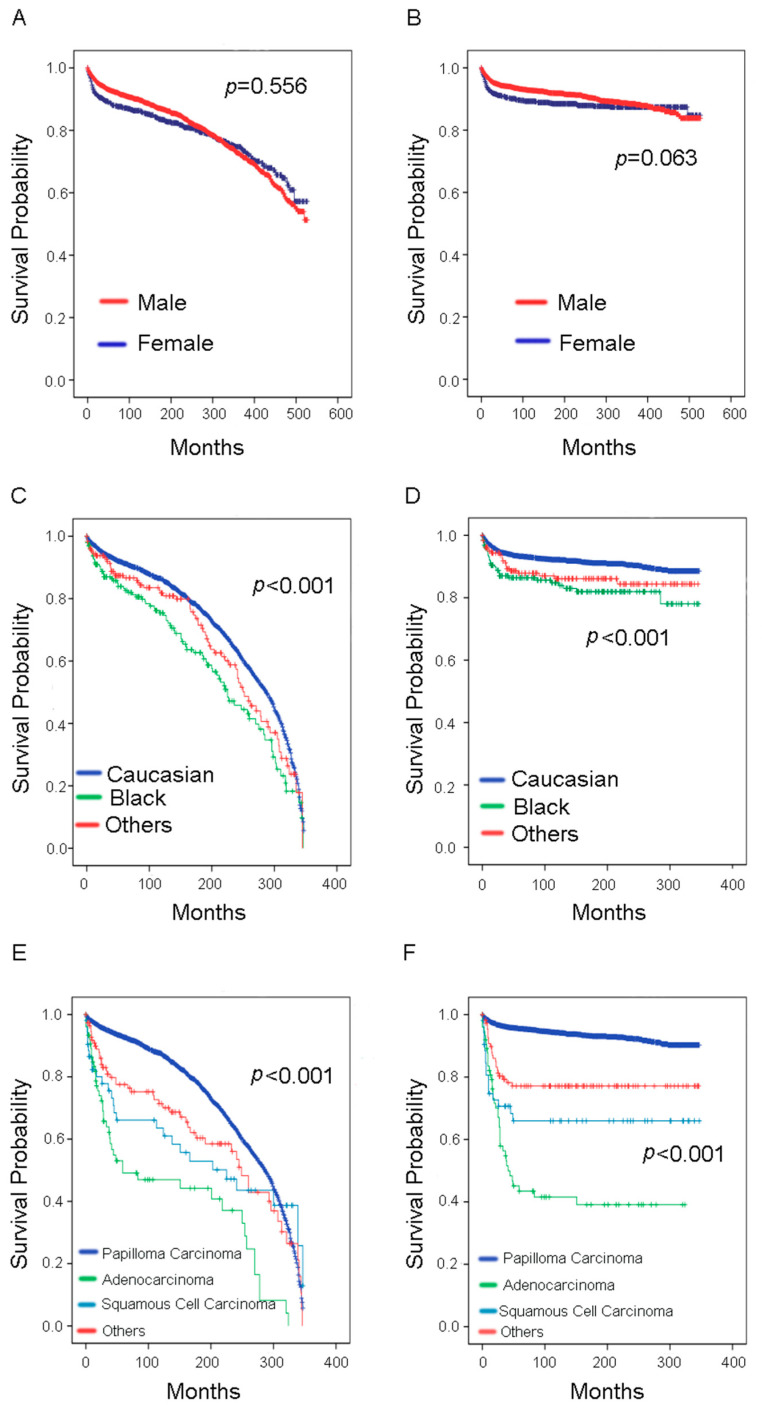
Survival analysis in younger bladder cancer patients with gender (**A**) OS, (**B**) CSS, race (**C**) OS, (**D**): CSS, and histological type (**E**) OS, (**F**) CSS. OS = overall survival; CSS = cancer-specific survival.

**Figure 4:**
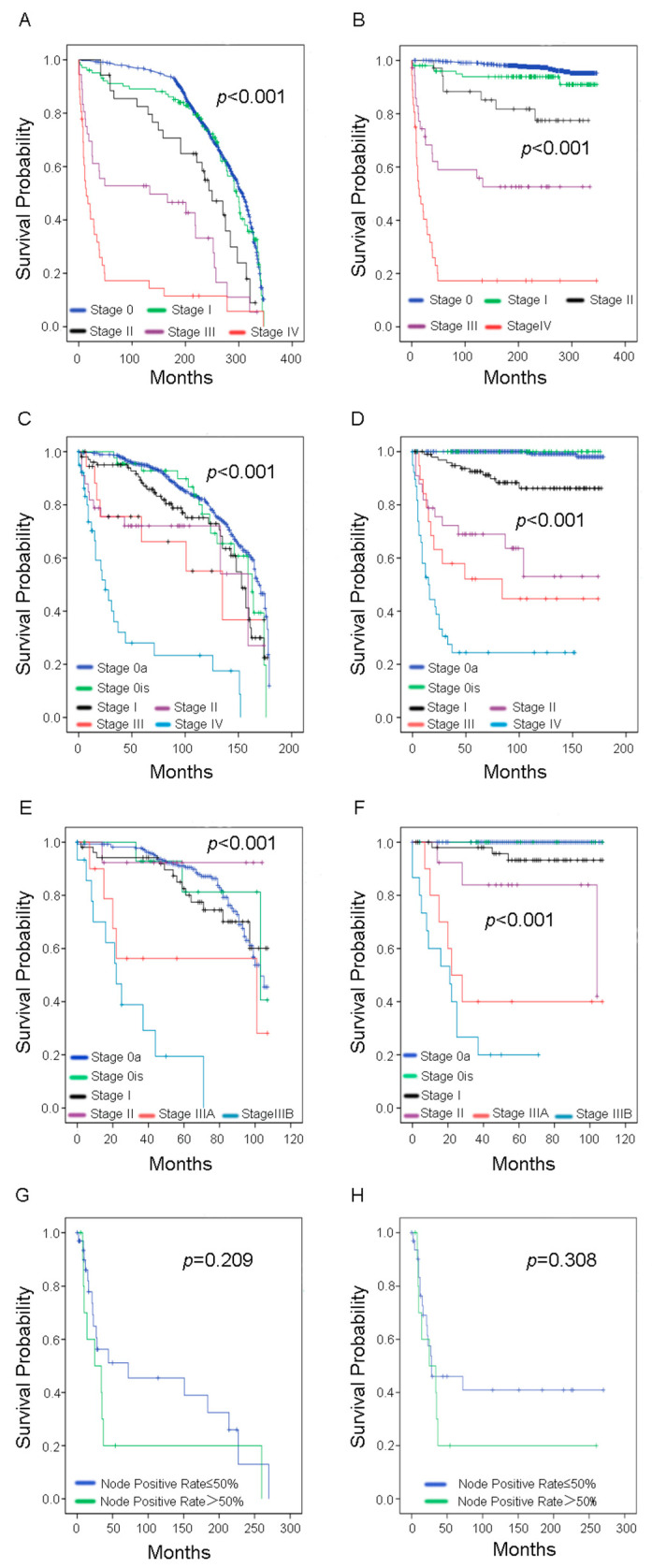
Survival analysis in younger bladder cancer patients with AJCC cancer stage based on each edition of the *AJCC* *Manual* (**A**,**B**) *AJCC Manual* 3rd edition; (**C**,**D**) *AJCC Manual* 6th edition; (**E**,**F**) *AJCC Manual* 7th edition) and node-positive rate (positive/examined) (**G**,**H**). Left column: overall survival; right column: cancer-specific survival.

**Figure 5:**
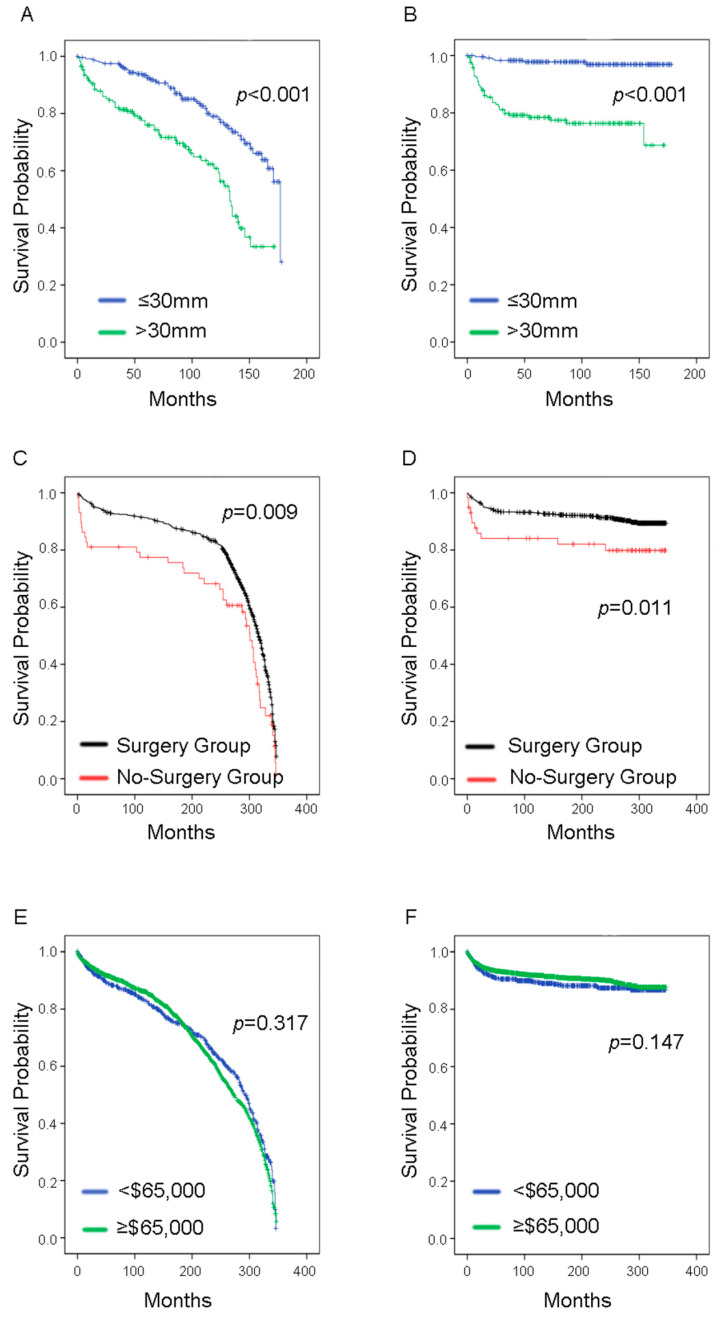
Survival analysis in younger bladder cancer patients with tumor size, treatment strategy, and household median income (**A**,**C**,**E**) overall survival; (**B**,**D**,**F**) cancer-specific survival.

The 10-year OS rate was 89.55%, while the 10-year CSS rate was 92.24%. As shown in Figure 2, Figure 3, Figure 4 and Figure 5, there were no statistical differences between OS and CSS regarding age, gender, node-positive rate, and household median income. However, there was a statistical difference in the year of diagnosis between OS. There were also statistical differences in race, histological type, cancer stage, tumor size, and surgery treatment groups between OS and CSS. In the race group, black people had lower survival rates compared with the other two groups (Caucasians and others). Regarding histological type, the papillary carcinoma group had the best survival rate, while the adenocarcinoma group had the worst survival rate. Regarding cancer stage, the survival rate decreased with increasing cancer stage. We found that survival curves were clearly separated from each other in CSS analysis when using the 6th edition *AJCC Staging System* (Figure 4D). The median tumor size was 30 mm; therefore, we separated patients into two groups according to their tumor size using 30 mm as the boundary. Patients in the tumors ≤30 mm size group had better OS and CSS than those in the >30 mm size group. Most of the patients underwent surgery in this study, and it was shown that those who had surgery had better survival rates than those who did not.

Univariate and multivariate Cox regression analyses were performed as shown in Table 1 and Table 2. There were significant differences in race, histological type, cancer stage, tumor size, and surgery treatment groups for predicting CSS using univariate Cox regression analysis. Given that the AJCC cancer stage classification system was updated several times between 1975 to 2018, we classified patients into three groups according to the edition of the *AJCC Cancer Stage Manual* used to perform the analysis. In multivariate Cox regression analysis, tumor size and cancer stage were the significant independent prognostic risk factors associated with CSS in bladder cancer in patients ≤40 years old (Table 2).

## 4. Discussion

In previous studies, there have been numerous prognostic factors identified in bladder cancer. Compérat et al. concluded that the main histological type was papillary carcinoma of the bladder in patients who were ≤40 years old [5]. Caione et al., through their training and validation cohort analysis, showed that patients who were <19 years old had a better prognosis [6]. Wang et al. demonstrated that age, gender, race, tumor grade, histological type, pathological stage, and surgical treatment were effective prognostic factors in bladder cancer [7]. He et al. showed that age at diagnosis, race, AJCC stage, and tumor size were independent prognosis factors for patients with urothelial bladder cancer who had undergone surgery [3]. Ma et al. collected data on patients who underwent cystectomy between 2004 and 2015 from the SEER database, and they showed that patients who were <50 years of age had a higher risk of lymph node positivity and superior outcomes [8]. However, the low incidence rate and small patient populations included in the previous studies limited further investigation of prognostic factors in bladder cancer patients ≤40 years old.

In our study, we collected data on the clinicopathological characteristics of patients with bladder cancer who were ≤40 years of age from the SEER database. We found that Caucasian patients diagnosed before 2000 had papillary carcinoma, an earlier cancer stage, a tumor ≤30 mm in size, and surgical treatment that showed a statistically better survival rate than patients in the control groups. Univariate Cox regression analysis showed that race, histological type, cancer stage, tumor size, and surgical treatment were independent prognostic factors in bladder cancer patients ≤40 years of age. However, in multivariate analysis, only tumor size and cancer stage were significant prognostic factors in bladder cancer patients ≤40 years of age. In bladder cancer patients ≤40 years of age, a larger tumor size and a more advanced stage were predictive of shorter CSS.

In our study, about 92% of patients had surgery. More than two decades before, Herr et al. showed that transurethral tumor resection (TURB) plus Bacillus Calmette-Guérin (BCG) resulted in a 10-year CSS rate of 75% in patients with non-muscle-invasive bladder cancer (NMIBC) [9]. Balar et al. showed promising antitumor activity of pembrolizumab monotherapy in patients with BCG resistance [10]. However, the survival data of adjuvant treatment with an immune checkpoint inhibitor in NMIBC are still unknown. Catto et al. performed a randomized controlled trial that showed that patients with high-risk NMIBC would have more clinical benefits after radical cystectomy compared with the TURB plus BCG group [11]. In our study, the 10-year CSS rate of included patients was 92.24%. This survival rate may be related to the low ratio of patients in the high-risk group and to the treatment that those patients received. We could not obtain data on treatment from the SEER statistics software, and this was one of the limitations of our study (see below).

Tilki et al. demonstrated the clinical association of AJCC cancer stage subclassification with prognosis, especially in patients with node-negative muscle-invasive bladder cancer [12]. Abdel-Rahman et al. demonstrated that, in predicting prognosis, it was essential to add substages III and IV in bladder cancer, which are described in the 8th edition *AJCC Manual*, especially for patients without neoadjuvant treatment [13]. In our study, we found that P values among all subgroups were significant and in agreement with the subgroups in the 6th edition *AJCC Manual* (Table 2); however, not all P values were significant for other groups. This may be because patients included in our study were not sub-classified, and this was the main reason there were differences in P values in each AJCC edition group; therefore, more studies are needed.

Our findings have several clinical implications. First, our results will fill the gap of the clinicopathological prognostic factors in bladder cancer patients ≤40 years old. Admittedly, because of the low incidence in patients ≤40 years old, statistical analysis has not been performed on a large sample size in this field. There have been studies that have analyzed prognostic factors in bladder cancer, particularly in unmarried men, adenocarcinoma bladder cancer patients, and patients who were treated with chemotherapy [14,15,16]. However, our study is the first that focused on patients ≤40 years old. Second, AJCC cancer stage and tumor size were identified as independent prognostic factors in these bladder cancer patients, which is in accordance with findings from previous studies that were limited to a younger population. However, there were also some differences with previous studies.

Our study had some limitations. First, we performed a retrospective study, and future prospective studies will be needed to validate our results. Second, we could not obtain the detailed treatment data for each patient from the database [17]; thus, this could not be analyzed in our study. Third, histology and immunohistochemical results could not be reviewed; therefore, expressions of prognosis-related genes were not evaluated. Fourth, an older cohort was not analyzed in our study as a comparison group, but we found better survival rates in the younger cohort compared with the older cohort (Supplementary Figure S1). Fifth, while overall and cancer-specific survival was evaluated in this study, we were not able to analyze other types of survival, making it a worthy subject of study for the future.

## 5. Conclusions

Our results suggest that tumor size and AJCC cancer stage in bladder cancer patients ≤40 years old are prognostic factors of survival. Tumors >30 mm in size and an advanced cancer stage were indicative of a poor prognosis in bladder cancer patients ≤40 years old, and long-term follow-up should be performed. Optimal management of these patients, however, will require more studies.

## Figures and Tables

**Table 1:** Patient characteristics and univariate Cox regression analysis predicting cancer-specific survival.

		Patient Characteristics			Univariate Cox Regression Analysis		
		Alive (N)	Dead (N)	*p* Value ^a^	*p* Value	HR	95% CI
Age at diagnosis	≤20 years	208	22	0.458	0.642	1.154	0.630–2.116
	>20 years	3195	364				
Year of diagnosis	≤2000 year	2203	271	0.174	0.153	1.223	0.928–1.613
	<2000 year	1201	114				
Gender	Female	922	120	0.279	0.064	0.814	0.655–1.012
	Male	2482	265				
Race	Caucasian	2961	305	0.347	0.001	1.388	1.147–1.678
	Black	226	48				
	Others ^b^	216	33				
Histological type	Papilloma carcinoma	3130	254	0.364	0.001	1.758	1.574–1.964
	Adenocarcinoma	52	59				
	Squamous cell carcinoma	88	29				
	Others ^c^	134	43				
*AJCC ^d^ Cancer Stage Manual* 7th Edition	0a	272	0	0.268	0.001	3.574	2.618–4.880
	0is	15	0				
	Ⅰ	50	3				
	Ⅱ	10	3				
	Ⅲ	4	6				
	Ⅳ	3	12				
*AJCC Cancer Stage Manual* 6th Edition	0a	531	3	0.266	0.001	2.814	2.417–3.276
	0is	45	0				
	Ⅰ	90	11				
	Ⅱ	21	12				
	Ⅲ	9	10				
	Ⅳ	11	29				
*AJCC Cancer Stage Manual* 3rd Edition	0	773	25	0.316	0.001	3.027	2.641–3.469
	Ⅰ	95	7				
	Ⅱ	28	7				
	Ⅲ	20	16				
	Ⅳ	7	30				
Node-positive rate	≤50%	17	16	0.626	0.316	1.547	0.659–3.629
	>50%	3	8				
Tumor size	≤30 mm	232	6	0.249	0.001	10.564	4.458–25.034
	<30 mm	132	38				
Surgery	Yes	635	69	0.458	0.013	2.244	1.187–4.242
	No	48	11				
Median income ^e^	<$65,000/year	595	73	0.272	0.148	0.812	0.613–1.076
	≥$65,000/year	1546	150				

HR = hazard ratio; CI= confidence interval. ^a^ Comparing each group. ^b^ Including Asian, Hispanic, Alaskan Native, and others unspecified. ^c^ Including uncommon histological type. ^d^ AJCC= American Joint Committee on Cancer. ^e^ Household median income.

**Table 2:** Multivariate Cox regression predicting cancer-specific survival-based AJCC stage.

		*AJCC Manual* 7th Edition (N = 203)			*AJCC Manual* 6th Edition (N = 380)			*AJCC Manual* 3rd Edition (N = 551)		
		*p* Value	HR	95% CI	*p* Value	HR	95% CI	*p* Value	HR	95% CI
Race	Caucasian	0.992	Ref	-	0.269	Ref	-	0.03	Ref	-
	Black	0.985	0.001	0.001–∞	0.107	0.181	0.023–1.442	0.235	1.527	0.760–3.069
	Others	0.900	1.111	0.215–5.751	0.945	1.049	0.271–4.065	0.01	3.138	1.594–6.174
Histological type	Papilloma carcinoma	0.448	Ref	-	0.602	Ref	-	0.093	Ref	-
	Adenocarcinoma	0.737	1.268	0.317–5.074	0.748	1.169	0.450–3.036	0.102	0.565	0.285–1.120
	Squamous cell carcinoma	0.985	0.001	0.001–∞	0.993	0.995	0.336–2.944	0.309	0.579	0.202–1.661
	Others	0.108	5.308	0.693–40.674	0.184	2.843	0.610–13.264	0.022	0.348	0.141–0.857
Tumor size	≤30 mm	-	Ref	-	-	Ref	-	- ^a^	-	-
	>30 mm	0.666	1.424	0.286–7.090	0.096	2.425	0.854–6.887	-	-	-
Cancer stage	0a/0	0.022	Ref	-	<0.001	Ref	-	<0.001	Ref	-
	0is	0.718	<0.001	0.000–3.472 × 10^−23^	0.983	<0.001	0.000–∞	- ^b^	-	-
	Ⅰ	0.892	<0.001	0.000–1.190 × 10^−64^	0.019	15.318	1.571–149.339	0.052	2.306	0.994–5.351
	Ⅱ	0.002	0.026	0.003–0.256	<0.001	83.302	9.883–702.177	<0.001	8.646	3.704–20.186
	Ⅲ	0.011	0.110	0.020–0.606	<0.001	118.849	13.370–1056.495	<0.001	33.407	16.747–66.639
	Ⅳ	0.107	0.294	0.066–1.304	<0.001	215.479	25.280–1836.379	<0.001	143.180	71.370–287.243

Ref = reference; ∞ = unmeasurable. ^a^ Tumor size was not documented before the year 2004. ^b^ There was not 0 as classification in the *AJCC Manual* 3rd edition.

## Data Availability

The data are publicly available from SEER database.

## Supplementary Materials

The following supporting information can be downloaded at: https://www.mdpi.com/article/10.3390/curroncol29020052/s1, Figure S1: Better survival in younger cohort.
