# Supplementary material for: Clinical Outcomes and Prognosis Analysis of Younger Bladder Cancer Patients
Source: Curr Oncol. 2022 Jan 28;29(2):578–88. doi: 10.3390/curroncol29020052 (PMC8870851; doi:10.3390/curroncol29020052)
Supplement: Supplementary file 1 [file curroncol-29-00052-s001.zip › curroncol-1454511-supplementary.pdf]

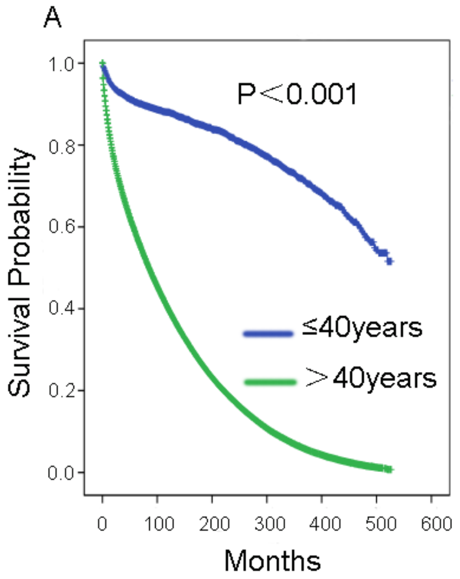

Supplementary Figure S1: Better survival in younger cohort. (A: overall survival; B: cancer specific survival; younger cohort: 3789 cases; older cohort: 218422 cases)
